# An Empirical Approach to Curriculum Mapping in Traditional Kampo Medicine Education in Japan: Practical Methodological Study

**DOI:** 10.2196/88430

**Published:** 2026-03-27

**Authors:** Yuya Masada, Masashi Ikuno, Karin Kato, Akihiko Ueda, Kaori Tsuyuki, Miki Ohtsuki, Kazuhisa Kaneda, Takuma Ohsuga, Maho Ueda, Neiko Ozasa, Miho Egawa, Akiko Tokinobu, Tomoko Miyoshi, Kiyoaki Tanikawa, Hitomi Kataoka

**Affiliations:** 1 Faculty of Medicine, Kyoto University, Kyoto, Japan; 2 Center for Medical Education and Internationalization, Faculty of Medicine, Kyoto University, Yoshida-Konoe-cho, Sakyo-ku, Building A, 2nd Floor, Kyoto, Japan, 81 0757539454; 3 Kampo Medicine Unit, Kyoto University Hospital, Kyoto, Japan

**Keywords:** traditional medicine, complementary and alternative medicine, CAM, curriculum mapping, evidence-based education, educational needs assessment

## Abstract

**Background:**

The integration of traditional and complementary medicine (T&CM) into modern medical education remains a global challenge. Kampo medicine, a Japanese traditional pharmacotherapy, is recognized in the *International Classification of Diseases, 11th Revision*, and is widely used; however, no structured methodology exists for efficiently designing curricula within limited time and resources. To address this gap, this study proposes a methodological framework—illustrated through Kampo medicine but generalizable to other forms of T&CM—for organizing and visualizing curricular content to guide educational needs assessment and curriculum design.

**Objective:**

The objective of this study was to develop and illustrate a reproducible mapping framework that integrates (1) real-world clinical utilization frequency and (2) the accumulation of biomedical evidence to inform educational prioritization and stepwise curriculum design for T&CM, using Kampo medicine as an exemplar.

**Methods:**

A mapping approach was developed based on two perspectives: frequency of use in clinical training and level of biomedical evidence. Twelve years of outpatient prescription data from Kyoto University Hospital were analyzed to identify the most frequently prescribed Kampo formulas. For the 10 most common formulas, PubMed searches were conducted to determine the number of randomized controlled trials. Data were integrated using hierarchical clustering and plotted along frequency–evidence axes to produce an educational priority map, which informed a stepwise curriculum design grounded in adult learning theory.

**Results:**

Prescription heat maps revealed substantial interdepartmental variation, and clustering identified distinct groups of formulas based on usage patterns. No statistically significant correlation was observed between prescription frequency and level of evidence (Pearson *r*=0.228, 95% CI −0.469 to 0.750; *P*=.53; Spearman ρ=0.498, 95% CI −0.308 to 0.926; *P*=.14). Integrating these two perspectives enabled interpretation of real-world prescription patterns and supported a transparent educational prioritization framework.

**Conclusions:**

This study presents a feasible and adaptable framework that links real-world clinical data with biomedical evidence to inform curriculum design in T&CM. Rather than prescribing specific content, the framework offers visual decision-making tools that align educational priorities with institutional practice patterns and can be readily adapted to complementary, alternative, and integrative medicine programs internationally.

## Introduction

In recent years, increasing international attention has been directed toward the challenge of integrating traditional and complementary medicine (T&CM) into modern medical education. The World Health Organization (WHO) has published a global strategy for traditional, complementary, and integrative medicine for 2025 to 2034, which aims to position traditional, complementary, and integrative medicine as an integral component of people-centered health systems [1]. A recent systematic review on integrating T&CM into health systems highlighted the need for reforms in medical education to support safe and effective integration, reinforcing the importance of strengthening undergraduate training in this area [2].

In East Asia, established systems such as Traditional Chinese Medicine, Korean Medicine (Hanbang), and Kampo medicine in Japan have developed not merely as complementary modalities but also as regionally rooted medical traditions institutionalized through education and practice [3].

Recent work in traditional East Asian medicine education has demonstrated a shift away from purely traditional apprenticeship models toward reconstruction using contemporary educational frameworks, such as competency-based education and structured curriculum design, highlighting parallel challenges and solutions in traditional medicine education [4]. A recent scoping review reported that complementary and alternative medicine teaching in undergraduate medical curricula is widely inconsistent in both content and assessment approaches, underscoring the need for more structured and evidence-based curricular design [5]. Curriculum alignment and mapping are widely used to ensure coherence and transparency in medical curricula and to identify gaps and redundancies; however, practical guidance on how to implement these processes remains limited [6]. Recent implementations of integrative medicine education for medical students have reported improvements in learners’ self-reported knowledge, attitudes, and course satisfaction; however, scalable approaches to prioritizing and sequencing curricular content remain an ongoing challenge [7].

Kampo medicine, Japan’s traditional system of pharmacotherapy, originated from classical Chinese medicine and was introduced via the Korean Peninsula in the 5th to 6th centuries. In 742, the Tang dynasty monk Jianzhen (Ganjin) brought Chinese medical texts and crude drugs to Japan, laying the foundations of Kampo practice [8-10]. Thereafter, Kampo evolved uniquely in response to Japan’s climate, culture, and constitution, while systematizing diagnostic and therapeutic principles with continued reference to Chinese classics [11,12]. Internationally, the WHO formally recognized Kampo as a traditional medical system in chapter 26 of the *International Classification of Diseases, 11th Revision* [13].

In Japan, Kampo medicines were officially integrated into the national health insurance system in 1976, thereby becoming broadly accessible to patients at relatively low cost [14,15]. Today, Kampo formulas are regulated by the Ministry of Health, Labor, and Welfare and are widely prescribed in routine clinical practice [16-19]. We previously reported on the extensive use of Kampo medicines at a university hospital, focusing in particular on their use in the Department of Anesthesiology and Pain Clinic [20]. More than 80% of Japanese physicians incorporate Kampo medicines into their practice, most often alongside Western medicine, highlighting their complementary role [21,22]. Importantly, the Model Core Curriculum for Medical Education in Japan, which provides the foundation for undergraduate training across all medical schools, explicitly includes the study of the fundamentals of Kampo medicine as one of its requirements [23]. Previous reports have suggested that the Japanese model of Kampo education could serve as a reference for traditional medicine curricula internationally, including in US medical schools [24]. Despite its historical background and widespread clinical use, undergraduate Kampo education in Japan remains constrained by limited curricular time, and the quantity and content of instruction vary across universities [25,26]. Although the scientific evidence base for Kampo has expanded with the accumulation of randomized controlled trials (RCTs) [10], an empirical and structured methodology for designing curricula that enables medical students to efficiently learn traditional medicine within limited time and resources has yet to be established.

In modern medical education, curricular priorities are often determined on the basis of disease prevalence and severity. For example, Machado et al [27] reported a curriculum design approach in Brazil that drew on mortality statistics and WHO burden of disease data (disability-adjusted life years) to align medical education with epidemiological impact. However, in traditional medicine education, the epidemiological frequency of diseases or conditions does not necessarily translate directly into curriculum design. Educational frameworks must instead focus on the distinctive modalities of intervention unique to traditional medicine [10]. In Kampo medicine, prescriptions are guided by *Sho*, a traditional diagnostic framework that classifies patients according to characteristic patterns of signs and symptoms rather than Western diagnostic categories, making the formula itself more closely aligned with the theoretical system. Consequently, the analysis of prescription frequencies lends itself to linking educational content with theoretical principles and offers a practical entry point for empirical approaches to curriculum design in traditional medicine education. In medical education, conducting a needs assessment is widely regarded as a foundational step in curriculum development, as it helps identify curricular goals and content priorities and align educational strategies with local context and available evidence [28]. Recent integrative work on theoretical models for undergraduate medical curriculum development has emphasized the importance of a situational analysis phase to align curricular objectives and content with the local context and stakeholders’ needs [29].

Although this case analysis is based on actual prescription data from Kyoto University Hospital, the objective is not to generalize or prescribe a specific set of formulas or teaching content across institutions. Rather, the emphasis is on the types of data, methods of integration, and visualization techniques that can serve as a methodological framework for educational needs assessment and curriculum design—one that is more broadly applicable to traditional medicine. In the context of constrained health care resources and compressed instructional time, the prioritization and visualization of curricular content may help safeguard the quality of traditional medicine education. Curriculum mapping has also been used to evaluate coherence and progression in competency-based curricula by visualizing how learning objectives form a coordinated learning spiral across courses and institutions [30]. Therefore, the aim of this study is to develop and illustrate a methodological framework for needs assessment and curriculum design in undergraduate traditional medicine education through the mapping and visualization of prescription data.

## Methods

### Study Overview

**Figure 1.**
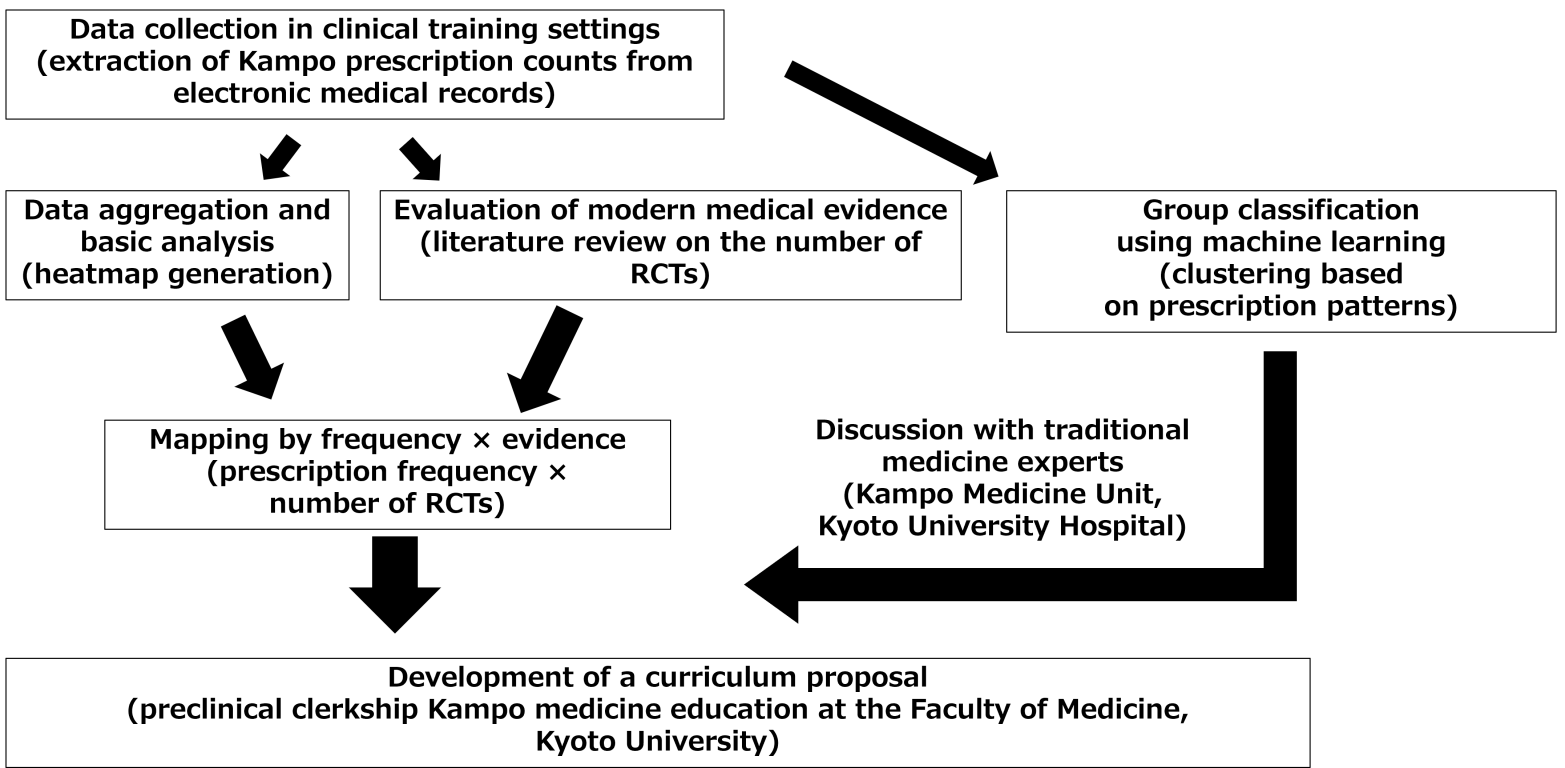
Overview of analytic steps for curriculum design in traditional medicine. This figure illustrates the process from collecting real-world data on traditional medicine use in clinical practice to integrating frequency, biomedical evidence, and machine learning–based clustering and finally applying these results to curriculum design. Items in parentheses represent the elements specifically analyzed in this study. RCT: randomized controlled trial.

This study presents a methodological framework for mapping and visualizing curricular content in traditional medicine education, focusing on two primary perspectives: frequency of use in clinical training settings and accumulation of biomedical evidence (measured by the number of RCTs). Clustering analyses were incorporated to extract structural insights, and the entire process was extended to the proposal of curriculum design. An overview of the analytic steps for traditional medicine curriculum development proposed in this study is shown in Figure 1.

### Data Sources

Prescription data on Kampo medicines under the national health insurance system were extracted from the electronic medical record system of Kyoto University Hospital for a 12-year period (April 2011 to March 2023). All clinical departments were included, and the number of prescriptions was recorded for each formula. Only Kampo products supplied by the same pharmaceutical company and continuously listed in the Kyoto University Hospital formulary throughout the 12-year study period were included; products newly introduced or discontinued during this period were excluded. In this study, “prescription count” refers to the number of times a Kampo formula was prescribed, regardless of dose or duration. Departments were aggregated according to electronic record system categories. Newly established or abolished divisions during the period were excluded, as were prescriptions recorded by the pharmacy for patient-brought medications. Subdivisions were consolidated under the higher-level department when applicable. Prescription frequency was operationalized as the number of prescription events recorded in the electronic medical record, irrespective of patient volume, dose, treatment duration, or repeated prescriptions for the same patient. This metric primarily reflects institutional prescribing activity and clinical workflow and should not be interpreted as a direct measure of unique patient exposure or the amount of hands-on clinical experience for individual medical students.

For the 10 most frequently prescribed formulas identified from the frequency data, an evidence evaluation was conducted through a literature review. PubMed searches were performed using each formula’s accepted English name (eg, Yokukansan, Yi-Gan San) combined with the keyword “randomized controlled trial.” Evidence strength was assessed based on the number of published RCTs. Inclusion criteria were original English-language articles published within the past 20 years that reported on the efficacy of the target formula. Exclusion criteria were articles without accessible full text (ie, unavailable through Kyoto University student accounts and institutional access) and studies not based on RCTs. Initial screening of titles and abstracts was conducted by one coauthor, followed by a full-text review and validation by another coauthor. In this study, the evidence indicator was operationally defined as the number of RCTs indexed in PubMed and published in English. This definition was adopted to ensure international comparability and accessibility for readers trained in biomedicine and does not represent the total accumulated evidence base for each Kampo formula.

### Data Analysis

Heat maps were generated to visualize the distribution of prescriptions by department. A pivot table of “Kampo formula × department” was created, and each cell contained the sum of prescriptions. The resulting prescription matrix was visualized using the *heatmap* function of Python Seaborn library. To improve readability, additional heat maps restricted to the top 10 departments and top 10 formulas were generated. All figures were produced using the Matplotlib and Seaborn libraries.

Hierarchical clustering was performed to analyze similarities in prescription patterns across departments. The “department × formula” matrix was standardized using *z* scores, followed by agglomerative clustering with Ward method and Euclidean distance. Departments were clustered based on their relative prescription profiles and visualized as dendrograms. Similar clustering was conducted on the formula dimension to highlight groups of prescriptions with shared patterns of departmental use. This approach enabled classification of departments based on the similarity of prescription patterns and provided descriptive structural context to inform educational discussions and curriculum design. Hierarchical clustering was implemented using the scipy.cluster.hierarchy module (Ward method, Euclidean distance).

Educational content was mapped by integrating two primary dimensions: frequency of clinical use (total prescriptions aggregated by department) and biomedical evidence (number of RCT reports identified through PubMed review). Hierarchical clustering of formulas based on prescription patterns was added to provide a structural context. Prescription counts, RCT counts, and clustering data were combined in a scatter plot.

### Statistical Analysis

Correlation analyses were performed between prescription counts and number of RCT reports to examine the relationship between clinical use and scientific evidence. Prescription counts were aggregated across departments for each formula. The Pearson correlation coefficient (*r*) was used to assess linear associations, and the Spearman rank correlation coefficient (*ρ*) was used to assess monotonic relationships. Analyses were performed using the *scipy.stats* module in Python, with *P* values of <.05 considered to indicate statistical significance. Effect sizes with 95% CIs were reported for correlation coefficients. Pearson CIs were calculated using Fisher *z* transformation, and Spearman CIs were estimated using percentile bootstrap resampling.

To provide a data-informed reference boundary (a pragmatic heuristic) for evidence, k-means clustering (k=2) was applied to RCT counts. A vertical reference line was drawn at the midpoint between the two centroids identified by clustering. Based on these integrated results, formulas were categorized from an educational perspective. The choice of k=2 dichotomized formulas into relatively evidence-rich and evidence-poor groups. This pragmatic classification reflects the educational purpose of determining a threshold in RCT availability rather than identifying multiple fine-grained clusters. Finally, as a case study, we constructed a curriculum design flow illustrating how such mapping could be integrated into traditional medicine education at Kyoto University.

**Figure 2.**
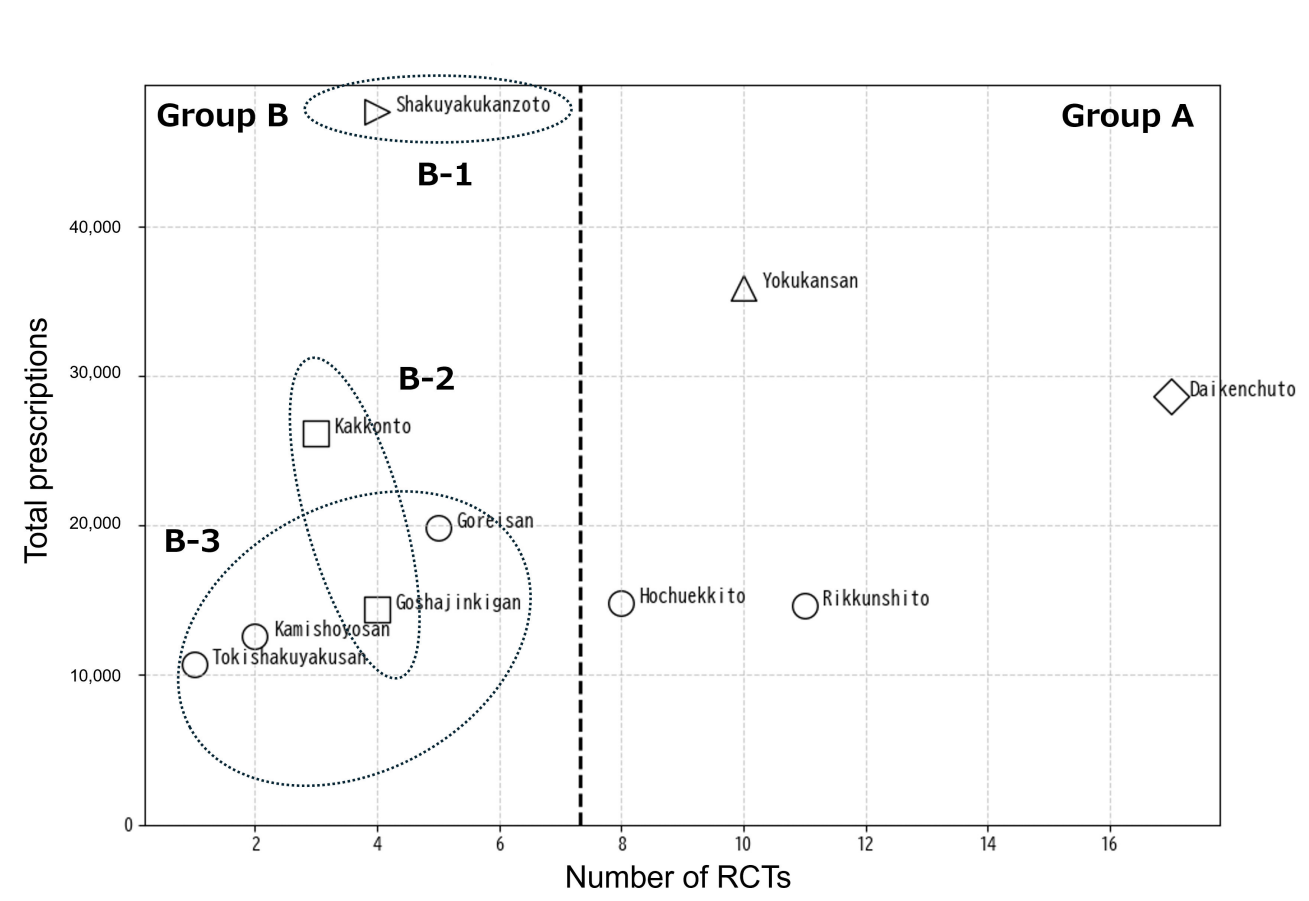
Mapping of Kampo formulas based on prescription frequency, evidence, and clustering information. Scatter plot showing total prescription counts (vertical axis) and number of randomized controlled trial (RCT) reports (horizontal axis). Marker shapes reflect cluster-based categorization. Group B formulas were subdivided into B-1 to B-3. A vertical dashed line indicates a data-informed reference boundary, defined as the midpoint between k-means centroids (k=2).

Given the small number of formulas included in the correlation analysis (n=10), robustness checks were performed using (1) permutation-based tests for correlation (10,000 permutations) and (2) leave-one-out analyses to assess sensitivity to influential formulas. For the evidence reference boundary shown in Figure 2, stability of 1-dimensional k-means thresholding (k=2) was evaluated by repeating the procedure across multiple random seeds (0‐199) and examining both centroid midpoint thresholds and resulting high/low group assignments. The k-means–derived reference boundary was compared with alternative cut points (median and 75th percentile of the RCT counts). The Kendall τ was calculated additionally as a rank-based correlation measure.

For hierarchical clustering, stability was assessed by repeating clustering under alternative preprocessing methods (eg, log1p transformation and row normalization), distance metrics (Euclidean, cosine, correlation), and linkage methods (Ward, average). Agreement with baseline clustering was quantified using the adjusted Rand index (ARI).

All analyses were performed in Python (version 3.12.2) using Pandas (version 2.2.2), NumPy (version 1.26.4), Matplotlib (version 3.9.0), Seaborn (version 0.13.2), SciPy (version 1.13.0), and Scikit-learn (version 1.5.0). The authors used ChatGPT (OpenAI, GPT-5, 2025) to assist with generation and refinement of Python scripts for data analysis and visualization. All code was reviewed, tested, and validated by the authors, who take full responsibility for the analyses and results presented in this study.

### Ethical Considerations

This study used routinely collected clinical prescription data from Kyoto University Hospital and involved secondary retrospective analysis without direct patient contact. The study protocol was reviewed and approved by the Kyoto University Graduate School and Faculty of Medicine Ethics Committee (approval number R3806). The requirement for informed consent was waived by the ethics committee because the study analyzed existing clinical data retrospectively, the study posed minimal risk, and all data were deidentified and analyzed in aggregate form. In accordance with institutional policy for secondary use of clinical data, an opt-out opportunity was provided through a publicly available disclosure notice. Patients could request exclusion of their data by contacting the responsible office listed in the notice. Data were stored and analyzed on secure institutional servers and were accessible only to the study team. No compensation was provided to participants because this study involved secondary analysis of existing data and imposed no additional burden on patients. All data were anonymized prior to analysis, and no personally identifiable information was accessible to the researchers. Data extraction and analysis were conducted in accordance with institutional data protection policies to ensure participant privacy and confidentiality.

## Results

### Prescription Frequency and Departmental Prescription Patterns

A total of 169,406 prescriptions were analyzed. To visualize the number of prescriptions by department and Kampo formula, heat maps were generated from pivot tables of “formula × department.” A subset focusing on the top 10 departments and top 10 formulas was extracted to highlight major prescription patterns (Multimedia Appendix 1). During the study period, the 10 most frequently prescribed Kampo formulas were shakuyakukanzoto, yokukansan, daikenchuto, kakkonto, goreisan, hochuekkito, rikkunshito, goshajinkigan, kamishoyosan, and tokishakuyakusan. The heat maps revealed not only the overall frequency distribution of each formula but also distinct interdepartmental differences in prescription patterns.

### Clustering of Departments Based on Prescription Patterns

To analyze similarities among departments, prescription matrices were standardized using *z* scores and subjected to hierarchical clustering (Ward method, Euclidean distance). Dendrograms demonstrated that the Pain Clinic, Neuropsychiatry, and Obstetrics & Gynecology departments exhibited distinctive prescription patterns and were classified as independent clusters, whereas most other internal medicine and surgical departments were grouped into a large shared cluster (Multimedia Appendix 2).

### Clustering of Kampo Formulas Based on Prescription Patterns

To evaluate similarities among formulas, hierarchical clustering of “formula × department” matrices (standardized by *z* scores, Ward method) was performed. Some frequently used formulas, including shakuyakukanzoto, yokukansan, and daikenchuto, demonstrated unique prescription patterns and formed independent clusters. The remaining formulas were grouped into 2 major clusters (Multimedia Appendix 3). Cluster 1 included 37 formulas with a total of 89,488 prescriptions, and Cluster 2 included 3 formulas with 23,870 prescriptions. Cluster 1 prescriptions were concentrated in pain clinics, neuropsychiatry, and obstetrics and gynecology, suggesting that they represented formulas familiar to these specialties. By contrast, the 3 formulas in Cluster 2 were used more broadly across multiple departments (Multimedia Appendix 4).

### Relationship Between Prescription Frequency and Evidence From Literature Review

For the 10 most frequently prescribed formulas, the number of RCTs identified in PubMed was compared with prescription counts (Multimedia Appendix 5). Correlation analysis using the Pearson correlation coefficient (linear association) and Spearman rank correlation coefficient (monotonic association) revealed no statistically significant correlations (Pearson *r*=0.228, 95% CI −0.469 to 0.750; *P*=.53; Spearman *ρ*=0.498, 95% CI −0.308 to 0.926; *P*=.14). Thus, among the most frequently prescribed formulas, no significant relationship was observed between frequency of clinical use and the number of RCT reports.

### Mapping by Frequency and Evidence Level

The top 10 formulas were mapped using 2 axes: prescription frequency and number of RCT reports. Complementary insights into group-level trends were obtained by overlaying formula clusters derived from prescription patterns (Figure 2). Formulas with both high prescription frequency and high evidence (daikenchuto, yokukansan, rikkunshito, hochuekkito) were classified as Group A. Formulas with high prescription frequency but relatively low RCT counts (shakuyakukanzoto, goreisan, kakkonto, goshajinkigan, kamishoyosan, tokishakuyakusan) were classified as Group B. For curriculum design purposes, Group B was further subdivided into 3 educational subgroups (B-1 to B-3), informed by the baseline clustering marker patterns. Shakuyakukanzoto was assigned to B-1; kakkonto and goshajinkigan to B-2; and goreisan, kamishoyosan, and tokishakuyakusan to B-3. This complementary classification allowed educational prioritization, even for formulas that were difficult to categorize by frequency or evidence alone. Marker shapes indicate clusters derived from hierarchical clustering based on prescription patterns (Multimedia Appendix 3). The subgroups B-1 to B-3 do not correspond directly to individual clusters in the dendrogram; rather, they represent educational subclassifications derived by integrating cluster membership (marker shape) with relative evidence levels along the RCT axis.

### Application of Mapping to Curriculum Design: Stepwise Learning Flow

**Figure 3.**
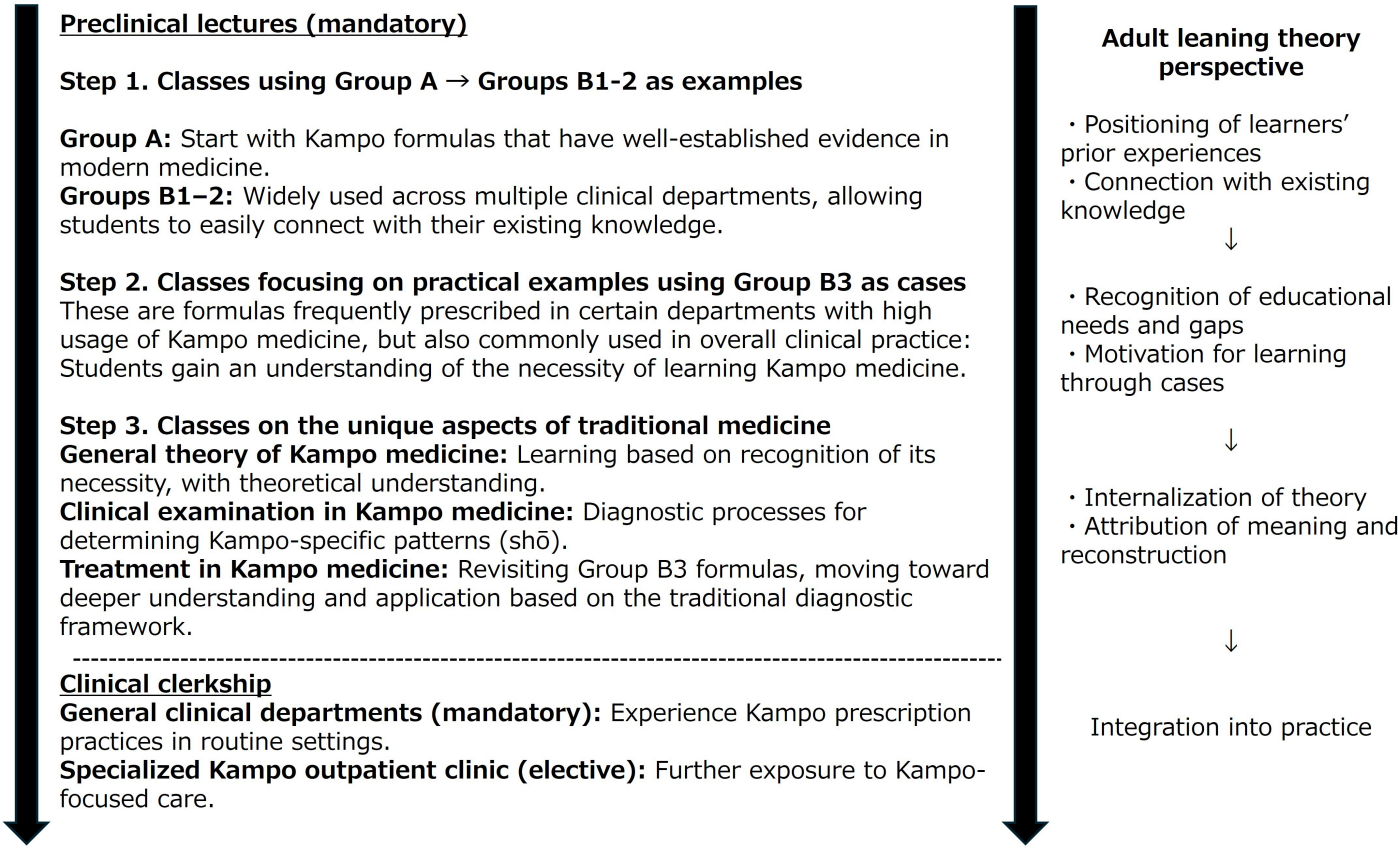
Stepwise Kampo education before clinical training and its alignment with adult learning theory. This figure illustrates the 3-step structure of preclinical Kampo lectures and its correspondence with adult learning processes: positioning learners’ experiences, recognizing knowledge gaps, fostering motivation through case examples, internalizing theoretical frameworks, and integrating learning into clinical practice.

Based on the integrated mapping of prescription frequency, evidence scores, and clustering, formulas were categorized from an educational perspective. As a practical application, a curriculum structure for preclinical Kampo medicine education was designed (Figure 3). This flow was developed in accordance with adult learning theory (Knowles), which emphasizes learners’ receptiveness and connection with prior knowledge [31].

Step 1 begins with Group A formulas, which have both high prescription frequency and relatively higher biomedical evidence (as indexed by PubMed RCT counts), making them well suited as introductory examples. Then, Groups B-1 and B-2 formulas are introduced. These are widely prescribed across multiple departments and easily connected to students’ prior biomedical knowledge, thereby facilitating smooth entry into Kampo learning.

In step 2, Group B-3 formulas are addressed. These are particularly prevalent in high-frequency prescription departments, such as pain clinics, neuropsychiatry, and obstetrics and gynecology. Case-based examples from these specialties emphasize their clinical relevance and highlight the need for further learning.

In step 3, the theoretical foundations of Kampo are presented, including diagnostic and therapeutic principles, while revisiting Group B-3 formulas. This reinforces understanding of the traditional diagnostic framework and facilitates the integration of knowledge into clinical applications.

### Sensitivity Analyses and Stability Checks

Given the small sample size for the correlation analysis (n=10 formulas), correlation estimates were imprecise and not statistically reliable across robustness checks. The Pearson correlation between RCT counts and total prescriptions was *r*=0.228 (*P*=.53; permutation *P*=.52), the Spearman correlation was *ρ*=0.498 (*P*=.14; permutation *P*=.14), and the Kendall *τ* was *τ*=0.360 (*P*=.15; permutation *P*=.18). Leave-one-out analyses showed that effect estimates varied (Pearson *r* range 0.108‐0.538; Spearman *ρ* range 0.310‐0.667; Kendall *τ* range 0.197‐0.500), consistent with limited power. Across 200 random initializations (seeds 0‐199), the 1-dimensional k-means (k=2) evidence boundary was identical (threshold=7.33; centroids=3.17 and 11.50) and yielded identical high/low assignments for all 10 formulas: Hochuekkito (8 RCTs), Yokukansan (10), Rikkunshito (11), and Daikenchuto (17) were classified as higher-evidence formulas in 100% of runs, whereas the remaining formulas (1‐5 RCTs) were classified as lower-evidence formulas in 100% of runs. Using alternative cut points changed the number of formulas classified as higher evidence (median cut point 4.5: 5/10; 75th percentile cut point 9.5: 3/10), indicating that borderline classifications depend on the chosen rule. The stability of hierarchical clustering varied by analytic settings. For departmental clustering, the top-level (k=2) partition was relatively consistent under log transformation and row normalization when using Ward linkage with Euclidean distance (ARI=0.81 and 0.76, respectively), whereas finer partitions and alternative metric/linkage choices showed lower agreement. For Kampo formula clustering, agreement across alternative preprocessing, metric, and linkage choices was generally low (ARI −0.05 to 0.37 across tested settings), indicating that specific formula-level cluster memberships should be interpreted as exploratory rather than definitive.

## Discussion

### Overview

In this study, we propose a methodological framework for organizing and visualizing educational content in traditional medicine curricula by integrating two perspectives—clinical use frequency and biomedical evidence—derived from real-world prescription data at a university hospital. This approach was then extended to curriculum design.

### Prescription Frequency and Departmental Prescription Patterns

Formulas prescribed with high frequency are those that medical students are most likely to encounter during clinical training. Advanced learning about these formulas allows students to apply knowledge directly in practice. Such experiences may enhance students’ perception of relevance (“what I learned was useful”), which in turn may stimulate self-directed learning. In this study, we did not explore departmental prescription biases in depth because our primary focus was the prioritization of limited preclinical instructional time and resources. Nevertheless, departmental prescription patterns likely reflect the specific patient populations and therapeutic cultures of each specialty. If curricular time for traditional medicine education can be secured during clerkships, educational interventions tailored to the prescription characteristics of each department may be meaningful. The prescription frequency and departmental data generated through the analytic flow presented here can also be applied to elective learning by offering individualized education aligned with students’ clinical placements.

Several limitations should be considered when interpreting prescription frequency. Because our analysis was based on aggregated prescription events, the results may overrepresent formulas used on an as-needed basis and underrepresent formulas prescribed for chronic or constitution-based management. In addition, prescription counts are influenced by documentation practices, departmental workflows, and institutional prescribing cultures [21], rather than reflecting standardized student exposure across clinical training. Accordingly, in this study, we use prescription frequency as a pragmatic indicator of institutional practice patterns relevant to educational needs assessment, not as a direct proxy for educational importance or learner exposure.

Several institution-specific factors should also be considered when interpreting the prescription data. Because this study was conducted at a university hospital, the case mix may differ from that of community-based settings, and common, uncomplicated diseases may be underrepresented [32]. In addition, the range of Kampo formulas available for prescription was constrained by the hospital formulary, which may limit the diversity of prescriptions observed. Furthermore, affinity for Kampo prescribing varies across clinical departments, reflecting differences in patient populations, clinical focus, and local prescribing cultures. These factors may influence prescription patterns and should be taken into account when interpreting the results. In particular, the prominent influence of the Pain Clinic in the clustering analysis likely reflects the clinical characteristics of this specialty, where Kampo medicines are frequently used for chronic pain management, symptom modulation, and adjunctive therapy alongside Western medicine. As a result, prescription volumes and formula diversity in the Pain Clinic tend to be higher than in many other departments, which may disproportionately affect clustering patterns.

Taken together, prescription frequency should be interpreted as a pragmatic indicator of institutional practice patterns for educational needs assessment. From an educational perspective, frequently encountered formulas can serve as an accessible entry point for learning. More complex Sho-based reasoning, constitutional diagnosis, and long-term management concepts can be progressively layered through guided instruction and expert facilitation.

### Clustering of Departments and Kampo Formulas

Hierarchical clustering based on prescription patterns was conducted for both departments and the Kampo formulas. The results revealed that certain specialties and formulas formed independent clusters, whereas many others were grouped together based on shared prescription patterns. This provides a visual and structural overview of the relationships between departments and formulas. However, this study had several limitations. First, clustering was based solely on prescription counts and did not capture prescribing intentions or department-specific treatment algorithms. For example, frequent use of a formula may reflect adherence to protocol or individual physician preference, distinctions not accounted for in this study. Second, prescription counts represent the total number of prescriptions and may include repeated prescriptions for the same patient or concurrent use of multiple formulas. In addition, decisions regarding the number of clusters and choice of distance metrics involve a degree of arbitrariness, which limits reproducibility and generalizability to other institutions. Prescription patterns may be influenced by patient demographics and case mix (eg, age, sex, and presenting symptoms), which were not statistically controlled in this study. Accordingly, cluster interpretation requires alignment with clinical and educational contexts rather than relying solely on numerical proximity. The value of clustering in this study lies not in identifying definitive prescription rules, but in providing a structural lens for reorganizing formulas and departments, thereby informing curriculum design.

### Evidence Evaluation Through Literature Review

For evidence evaluation, we used the number of RCTs published in English and indexed in PubMed, a widely used international database. This approach has limitations because Japanese-language publications and studies published in domestic journals were not captured. However, the intent was to present indicators that are comprehensible and relatable to medical students trained in biomedicine while simultaneously offering perspectives that could inform curriculum design in diverse educational settings worldwide. For this reason, the evidence evaluation was restricted to English-language PubMed-indexed RCTs.

Accordingly, the evidence level of some widely used Kampo formulas may be underestimated in this mapping, which could influence their classification into lower-evidence categories. Therefore, the evidence axis should be interpreted as reflecting the availability of international, English-language biomedical evidence rather than overall scientific validity. In addition, characteristics of RCTs for Kampo medicines warrant consideration. In Japan, many commonly prescribed Kampo formulas have long been covered by the national health insurance system and widely incorporated into routine clinical practice [14,15]. As a result, there may be limited commercial or regulatory incentive to conduct large-scale RCTs for these established formulas, whereas newer or less widely used formulas may be more likely to be investigated in formal clinical trials [33]. This structural feature of the evidence base should be taken into account when interpreting the distribution of RCT counts across formulas.

In terms of student comprehensibility, not only proof of efficacy by RCTs but also mechanistic understanding is relevant. However, establishing objective criteria for mechanistic elucidation is challenging. Given our aim to develop a feasible methodology applicable to a wide range of traditional medicine curricula, we restricted our evaluation to RCTs. In practice, expert consultation remains necessary when interpreting data and determining the specific educational content for curriculum development.

### Mapping by Frequency and Evidence

Our analysis revealed that several formulas were frequently prescribed despite limited RCT-based evidence, highlighting a clear gap between clinical practice and biomedical evidence. While bridging this “evidence gap” through further research is undoubtedly valuable, our findings suggest an additional perspective: these gaps may represent educational opportunities in which traditional medicine frameworks can be explicitly addressed. Rather than excluding formulas with limited biomedical support, traditional medicine education can help students appreciate the theoretical and cultural contexts underlying such practices. This provides unique learning that complements biomedical training rather than duplicates. Thus, the mapping methodology presented here offers a framework for reexamining the selection and sequencing of curricular content.

The evidence threshold used to distinguish higher- and lower-evidence categories was derived from k-means clustering (k=2) and represents a relative, data-dependent boundary within this dataset. Formulas located near the threshold may shift categories if analytic settings or datasets change. Accordingly, these groupings should be interpreted as pragmatic tools for relative educational prioritization rather than as absolute classifications of therapeutic validity or efficacy.

Several statistical limitations should be acknowledged. First, the correlation analysis was based on a small number of formulas, which limits statistical power and precision. Consistently, the 95% CIs around the correlation coefficients were wide, underscoring the limited precision of the correlation analysis. Second, clustering results and the evidence threshold depended on methodological choices such as distance metrics, linkage methods, and threshold settings. Consequently, group labels should be interpreted as relative and pragmatic categorizations within this dataset rather than fixed or universally applicable classifications.

### Curriculum Design Informed by Mapping

The proposed curriculum design incorporated both the use frequency and evidence level, supplemented by clustering-derived group characteristics, to structure a stepwise learning flow. Beginning with formulas that are strongly evidence-based or easily connected to students’ biomedical knowledge, and then progressing to formulas less easily explained within biomedical frameworks, aligns with adult learning theory, including processes of recognizing learning needs, internalizing theoretical perspectives, and transferring knowledge to clinical practice [31]. In medical education, self-directed learning has been reported to be an effective learning strategy compared with traditional didactic approaches, supporting our emphasis on designing learning sequences that foster learner autonomy and engagement [34].

**Figure 4.**
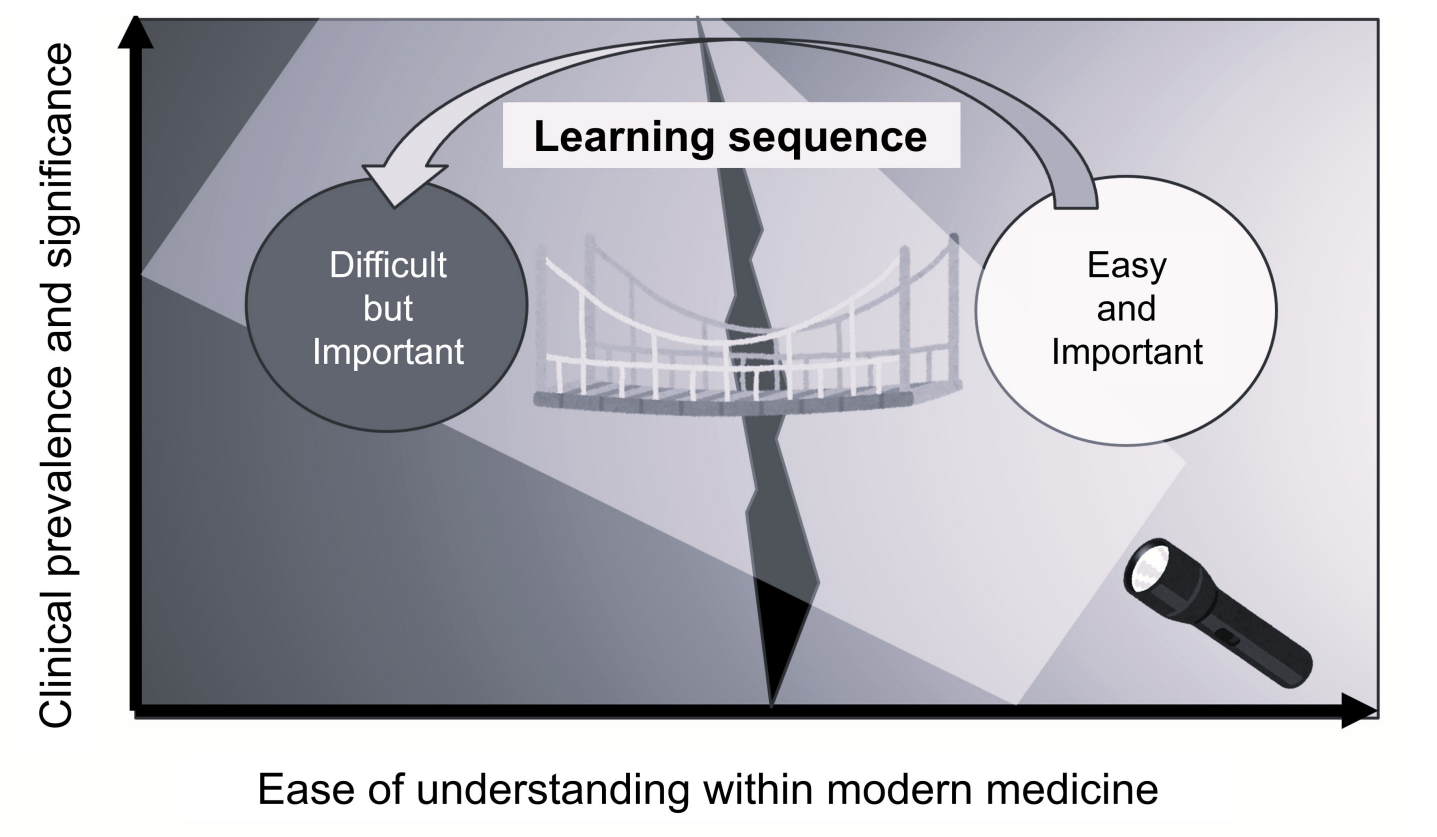
Conceptual diagram of strategic mapping in traditional medicine education. This figure presents a conceptual diagram that visualizes priority areas in traditional medicine education based on a 2-axis framework: affinity with biomedical knowledge (horizontal axis) and clinical importance or frequency (vertical axis). The intent is to begin instruction with the “Easy and Important” domain (content that is both accessible to learners and clinically significant) and then gradually progress to the “Difficult but Important” domain, thereby bridging existing educational gaps. By highlighting the suggested learning sequence and positioning of educational content, this framework supports strategic curriculum design that makes efficient use of limited educational efforts.

In Figure 4, the horizontal axis represents learners’ relative ease or difficulty in understanding Kampo concepts within a biomedical framework. Importantly, this “difficulty” should be understood as arising from at least 2 distinct factors. One factor is the limited availability of clearly articulated biomedical mechanisms, which may be partially reflected by the volume of RCT-based evidence. The other factor is the conceptual and terminological gap between Kampo medicine and modern biomedicine, as Kampo relies on its own diagnostic and therapeutic framework, including Sho-based reasoning [35], that is unfamiliar to many biomedical learners.

Accordingly, Figure 4 is intended as a conceptual guide for curriculum design rather than a direct mapping of RCT volume to learning difficulty. From an educational perspective, introducing frequently encountered formulas with relatively higher biomedical interpretability may lower the initial learning barrier. Learners can be progressively supported in engaging with Kampo-specific terminology, diagnostic concepts, and theoretical frameworks through structured instruction and expert facilitation.

This study was based on data from a single institution, and the specific flow proposed here should not be directly applied to other settings. However, given that Kyoto University Hospital is a multispecialty teaching hospital and a leading educational institution in Japan, the analytic process demonstrated here may serve as a feasible and flexible methodological framework for needs assessment and curriculum design at other institutions.

Our sensitivity analyses highlight that correlation, thresholding, and clustering results depend on analytic choices and are limited by sample size. With only 10 formulas, correlation results remained statistically non-significant across permutation and leave-one-out analyses, and effect estimates were imprecise. Although the k-means–derived reference boundary was stable across random initializations in this dataset, alternative reasonable cut points (eg, median or upper-quartile thresholds) lead to different classifications for borderline formulas, underscoring that the boundary should be treated as a heuristic rather than an objective standard. In addition, hierarchical clustering—particularly at the formula level—showed limited stability across preprocessing, distance metrics, and linkage methods. We therefore use clustering primarily as a descriptive visualization tool to highlight heterogeneity in prescribing patterns rather than as a definitive taxonomy.

In summary, the purpose of this study was not to prescribe a fixed set of educational content but to present a process for creating and utilizing visual decision-making tools that can be flexibly adapted to institutional educational philosophies and clinical contexts. This approach may provide a practical and generalizable framework for bridging educational practices with clinical reality in traditional medicine education.

### Conclusion

In this study, we organized and visualized educational content in traditional medicine by integrating three components: prescription frequency, level of biomedical evidence, and clustering information based on prescription patterns derived from real-world data at a university hospital. Through this mapping methodology, existing educational gaps can be structurally identified, and the approach can be applied to the stepwise arrangement of learning content. To effectively promote traditional medicine learning within limited educational resources, curriculum design should introduce students to “Easy and Important” content (material that is readily accessible and easily connected to biomedical knowledge) while gradually bridging toward the “Difficult but Important” areas that may be less intuitive for learners but highly significant in clinical practice.

The conceptual diagram presented in this study (Figure 4) focuses on areas insufficiently addressed by biomedical-based curricula, and by visualizing the positioning of “content easily accessible to learners” versus “content requiring specialized education,” it proposes a direction for educational construction that strategically and efficiently bridges these domains. We believe that this methodology serves as a practical framework for supporting the structural design of education and holds potential for application in other institutions and disciplines.

## Supplementary material

10.2196/88430 Multimedia Appendix 1 Heat maps of Kampo prescription counts by department and formula. (A) Heat map showing the overall distribution of prescriptions across departments (vertical axis) and formulas (horizontal axis). (B) Heat map restricted to the top 10 departments and top 10 formulas. Color intensity reflects prescription frequency, clearly illustrating formula-specific trends across departments.

10.2196/88430 Multimedia Appendix 2 Hierarchical clustering of departments based on Kampo prescription patterns. Departments with similar prescription profiles were clustered together. The Pain Clinic, Neuropsychiatry, and Obstetrics & Gynecology formed distinct independent clusters, while most other departments clustered together due to similar prescription patterns.

10.2196/88430 Multimedia Appendix 3 Hierarchical clustering of Kampo formulas. Some formulas (eg, yokukansan, shakuyakukanzoto, daikenchuto) formed distinct independent clusters, while the majority were grouped into three clusters based on departmental prescription distributions.

10.2196/88430 Multimedia Appendix 4 Distribution of prescriptions in Clusters 1 and 2 across departments. Cluster 1 formulas were prescribed predominantly in three departments (Pain Clinic, Neuropsychiatry, Obstetrics & Gynecology), while Cluster 2 formulas showed broader cross-departmental use.

10.2196/88430 Multimedia Appendix 5 Relationship between prescription frequency and randomized controlled trial (RCT) counts. The number of RCT reports (evidence scores) for the top 10 formulas identified by PubMed review is shown against prescription frequency.

## Abbreviations

ARI: adjusted Rand index
RCT: randomized controlled trial
T&CM: traditional and complementary medicine
WHO: World Health Organization
