# Supplementary figures and images for: An Empirical Approach to Curriculum Mapping in Traditional Kampo Medicine Education in Japan: Practical Methodological Study
Source: JMIR Form Res. 2026 Mar 27;10:e88430. doi: 10.2196/88430 (PMC13026446; doi:10.2196/88430)

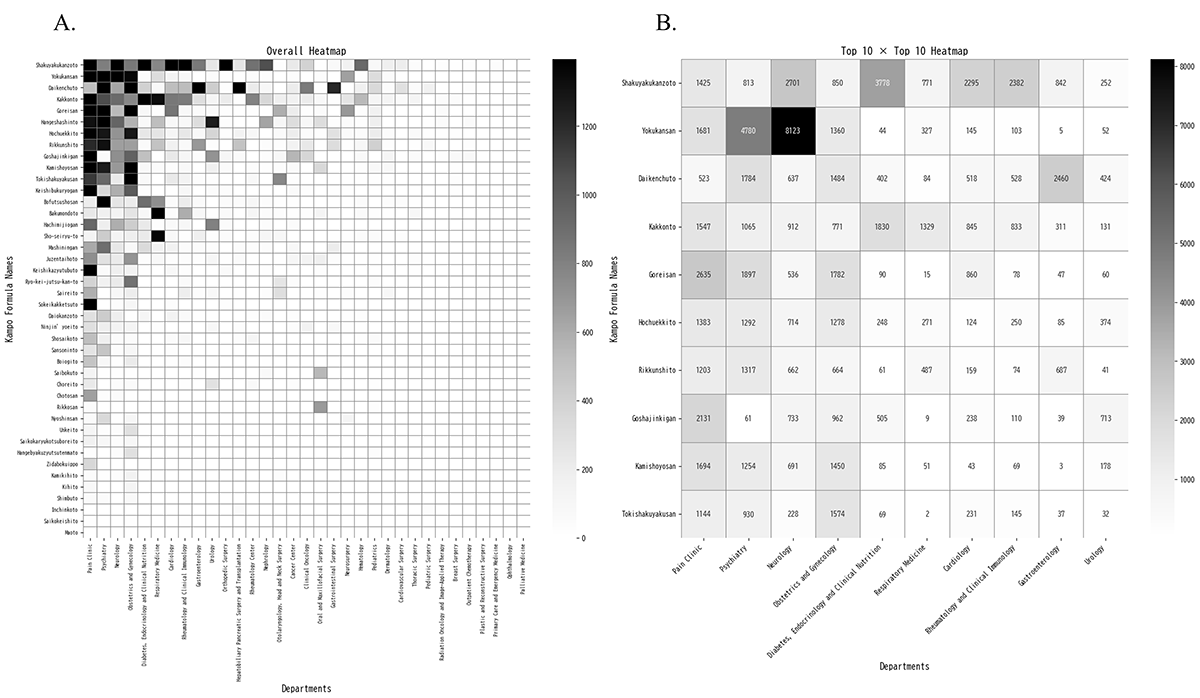

Supplement: Multimedia Appendix 1 [file formative-v10-e88430-s001.png]

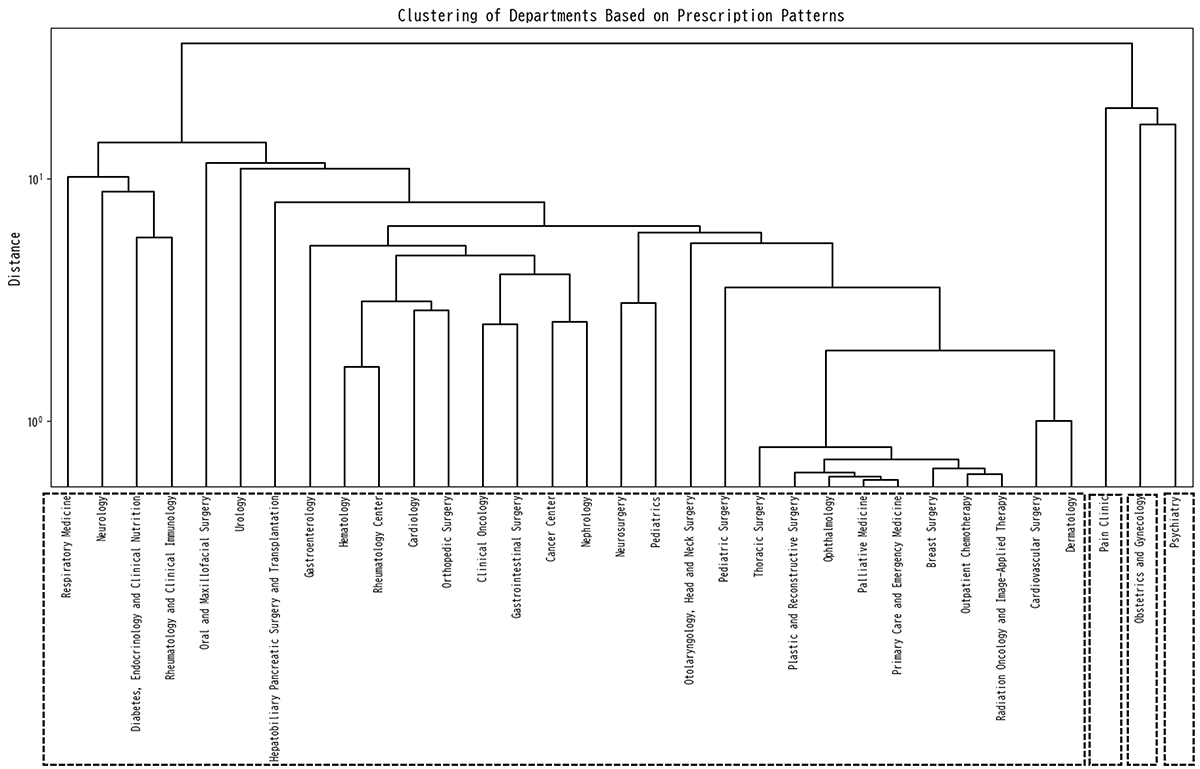

Supplement: Multimedia Appendix 2 [file formative-v10-e88430-s002.png]

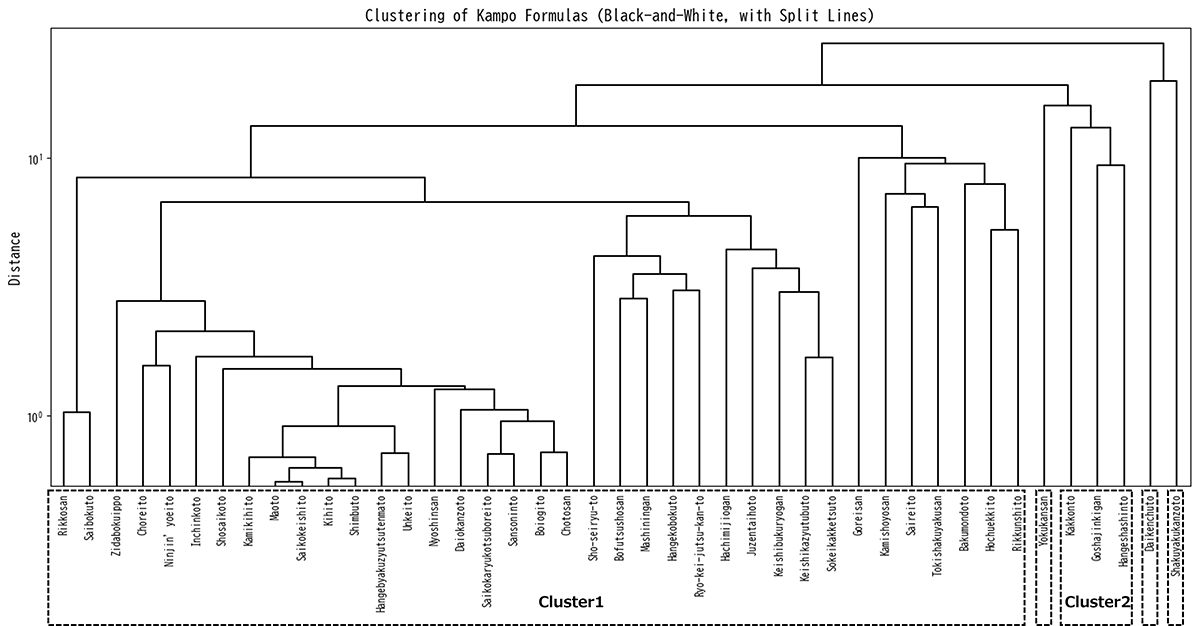

Supplement: Multimedia Appendix 3 [file formative-v10-e88430-s003.png]

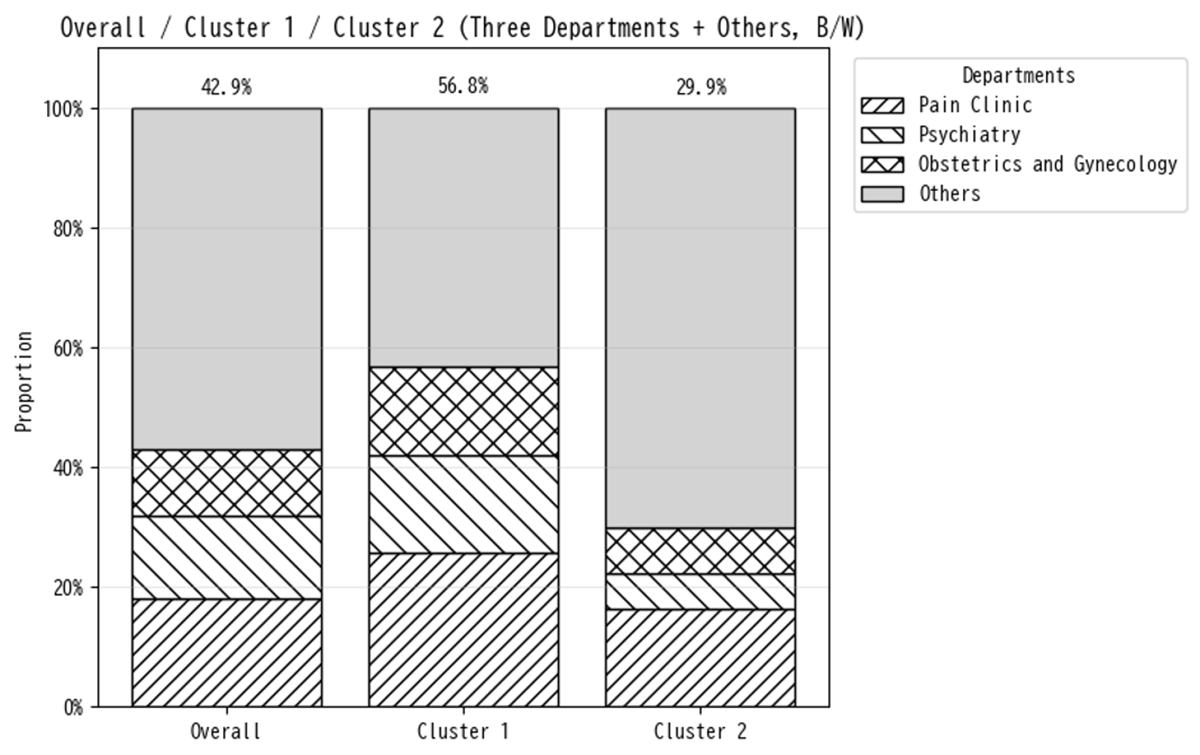

Supplement: Multimedia Appendix 4 [file formative-v10-e88430-s004.png]

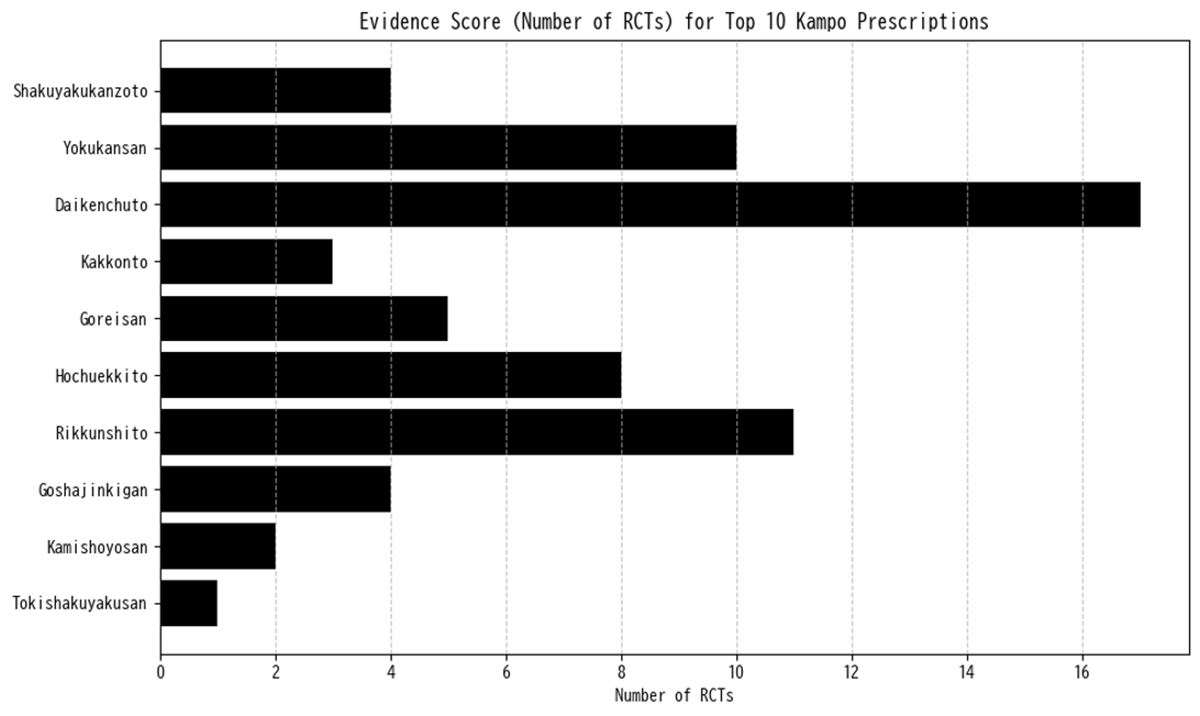

Supplement: Multimedia Appendix 5 [file formative-v10-e88430-s005.png]
